# Population Aging and Heat Exposure in the 21st Century: Which U.S. Regions Are at Greatest Risk and Why?

**DOI:** 10.1093/geront/gnad050

**Published:** 2023-04-28

**Authors:** Deborah Carr, Giacomo Falchetta, Ian Sue Wing

**Affiliations:** Department of Sociology and Center for Innovation in Social Science, Boston University, Boston, Massachusetts, USA; International Institute for Applied Systems Analysis, Vienna, Austria; Euro-Mediterranean Center on Climate Change, Venice, Italy; Department of Earth and Environment, Boston University, Boston, Massachusetts, USA

**Keywords:** Age structure, Climate change, County-level analyses, Geographic differences

## Abstract

**Background and Objectives:**

The co-occurring trends of population aging and climate change mean that rising numbers of U.S. older adults are at risk of intensifying heat exposure. We estimate county-level variations in older populations’ heat exposure in the early (1995–2014) and mid (2050) 21st century. We identify the extent to which rising exposures are attributable to climate change versus population aging.

**Research Design and Methods:**

We estimate older adults’ heat exposure in 3,109 counties in the 48 contiguous U.S. states. Analyses use NASA NEX Global Daily Downscaled Product (NEX-GDDP-CMIP6) climate data and county-level projections for the size and distribution of the U.S. age 69+ population.

**Results:**

Population aging and rising temperatures are documented throughout the United States, with particular “hotspots” in the Deep South, Florida, and parts of the rural Midwest. Increases in heat exposure by 2050 will be especially steep in historically colder regions with large older populations in New England, the upper Midwest, and rural Mountain regions. Rising temperatures are driving exposure in historically colder regions, whereas population aging is driving exposure in historically warm southern regions.

**Discussion and Implications:**

Interventions to address the impacts of temperature extremes on older adult well-being should consider the geographic distribution and drivers of this exposure. In historically cooler areas where climate change is driving exposures, investments in warning systems may be productive, whereas investments in health care and social services infrastructures are essential in historically hot regions where exposures are driven by population aging.

Climate change has dire consequences for older adults’ health and well-being (Meade et al., 2020). Global increases in the frequency, intensity, and duration of extreme heat spells pose the most direct threat, given older adults’ heightened susceptibility to hyperthermia and common health conditions worsened by heat exposure such as cardiovascular disease (Khatana et al., 2022). These threats are exacerbated for older adults who are socially isolated, live in substandard housing with inadequate cooling systems, or lack financial resources to adapt their homes to heat extremes (Gamble et al., 2013). The multiple heat-related deaths of Florida nursing home residents following an extensive power outage during Hurricane Irma in 2017 starkly reveal how climate change-driven increases in ambient temperatures threaten older adults (Skarha et al., 2021).

Despite extensive literature documenting the individual-level effects of extreme heat on older adults’ health and mortality risk, gerontological researchers have paid less attention to older adults’ heat exposure at the *population level* (Gamble et al., 2013; Meade et al., 2020). Two co-occurring trends—population aging and climate change—may create a context in which particular U.S. regions become “hotspots” with *both* increasing concentrations of older adults and high-temperature extremes. These hotspots will be at elevated levels of population-level heat exposure, placing high demands on local governments to develop appropriate infrastructures and response systems (Dahl et al., 2019).

We apply measures and methods widely used in environmental sciences to estimate (a) current (2020) and projected change (2020–2050) in the population of adults age 69+ in the 3,109 counties in the 48 coterminous United States; (b) contemporary (1995–2014) levels of and projected changes in (by 2050) heat exposure, measured as cooling degree days (CDDs) and 95th percentile of daily maximum temperature (TMax_95_); (c) county-level variations in population heat exposure, measured as the co-occurrence of both population aging and the two climate measures (CDD, TMax_95_); and (d) the relative contributions of population aging, population growth, and climate change to population heat exposure in the contiguous 48 U.S. states and nine Census geographic divisions. The analyses will pinpoint regions most susceptible to population-level extreme heat exposure and will provide useful information to policy makers and practitioners developing long-term strategies to protect older residents from heat extremes.

## Background

### Climate Change and Older Adult Well-Being

Since the 1960s, the frequency of extreme heat and weather events in the United States and worldwide has increased dramatically (Dahl et al., 2019). The period between 2015 and 2022 recorded the highest average temperatures in history (World Meteorological Organization, 2023). Rising temperatures are driven largely by emissions of heat-trapping greenhouse gasses caused by human activity (Vose et al., 2017). The harmful health consequences of heat extremes are well established, with older adults especially vulnerable (Ayalon et al., 2021; Meade et al., 2020). Age-related biological changes reduce older adults’ capacity to thermoregulate (Sajjad et al., 2022). Medications commonly taken by older adults can further intensify their vulnerability to heat exposure (Millyard et al., 2020). Anticholinergic medications, used to treat health conditions including chronic obstructive pulmonary disorder, reduce one’s capacity to sweat and thermoregulate (Westaway et al., 2015). Beta-blockers and diuretics can cause side effects that intensify heat-related symptoms such as dehydration (Meade et al., 2020). Age-related physical, cognitive, and sensory impairments may undermine an older adult’s capacity to prepare for and respond to heat extremes (Davies & Bhutta, 2022).

### Population Aging and Heat Exposure

Gerontological research on climate change and its impacts on older adults largely emphasizes *individual-level* sources of vulnerability and resilience, such as physiological factors that heighten sensitivity to temperature extremes, and personal resources that enable one to manage the impacts of temperature extremes, such as high-quality housing with sufficient cooling systems, supportive networks, and cognitive capacities that enable effective risk assessment and planning (Gamble et al., 2013; Meade et al. 2020). Yet vulnerability and resilience also operate at a *population level*; differential exposure to extreme heat varies across regions of the United States, affecting communities’ capacities to create responsive infrastructures. Social and economic factors, such as the final resources available for infrastructure investments, the political and public will to implement carbon neutrality goals, and the efficacy of local warning systems, influence a region’s capacity to adapt to rising temperature extremes (Howe et al., 2015). Locations with large and growing populations of older adults *and* rising heat extremes are likely to face the most urgent demands for effective adaptation (Jones et al., 2015).

Population aging is arguably the most significant force impelling the need for such adaptations. (Other population characteristics including education, poverty status, and race/ethnicity also affect an area’s vulnerability and capacity to respond to climate change; Environmental Protection Agency, 2021.) In 2020, adults ages 65+ accounted for 17% of the U.S. population, with a projected increase to 25% by 2060. U.S. states and regions are not aging uniformly, however. Due to population processes including differential birth rates, migration, and aging-in-place, older adults are disproportionately concentrated in particular regions. States with the oldest population structures, where one-fifth of residents are ages 65+, include retirement magnets such as Florida, as well as rural New England and Rust Belt states marked by aging-in-place, such as Maine and West Virginia. By contrast, just 11% of Utah residents are age 65+ due to high birth rates. Variation at the county level is even wider, ranging from 15% in Lee County, VA, to more than 58% in Sumter County, FL—home to The Villages, the nation’s largest retirement community (U.S. Census Bureau, 2020b). In addition to being the “oldest” state in the nation, Florida is particularly affected by extreme climate and weather events, exposing large numbers of older adults to risk (Gamble et al., 2013).

Identifying regions of the U.S. projected to become “hotspots” with growing concentrations of both older adults and heat exposures requires the use of sophisticated measures and methods developed by spatial and environmental scientists (Jones et al., 2015; Striessnig et al., 2019). These approaches are ideally suited for addressing an urgent question in social gerontology: In which U.S. counties and states are older adults experiencing the greatest exposure to extreme temperatures, and how will that exposure change in the future? Projecting changes in older adults’ heat exposure is critical for adaptation planning and implementation. Yet refining what these efforts should entail also depends on the *mechanisms* driving exposure. Climate change research identifies three key drivers of a region’s future heat exposure: population growth, changes in the population’s age structure, and rising temperatures. The relative contributions of each driver may differ across regions, requiring tailored interventions (Jones et al., 2015).

### The Present Study

Our goals are to describe the contemporary exposure of older U.S. adults to extreme heat and to project exposure at midcentury (2050); identify geographic regions that are most vulnerable to this exposure; and identify the main drivers of increased exposure. Areas projected to experience both population aging and rapidly warming climates are at the greatest risk of population-level heat exposure. Thus, analyses require fine-grained population aging and climate data across meaningful geographic units. To document population aging, we use county-level estimates of the size and geographic distribution of the age 69+ population in the 3,109 contiguous U.S. counties in 2020 and projected change from 2020 to 2050. To estimate heat exposure, we use measures of daily average temperatures, which capture *cumulative heat exposure* over the year, and the 95th percentile of daily maximum temperatures, which capture *acute exposure to heat extreme*s. We focus on both chronic and acute measures because each poses distinctive risks to older adults. Chronic exposure to temperatures above 77°F (25°C) in conjunction with high humidity can cause heat stress (Asseng et al., 2021). Even brief exposures to very high temperatures exceeding 95°F (35°C) with high humidity can threaten older adults’ health, with increases in the frequency and duration of high temperatures intensifying these risks. We use these measures to estimate contemporary and future heat exposure of older adults and county-level variation in that exposure and estimate the relative contributions of population aging, population growth, and climate change to older adults’ heat exposure, at the state and Census region level.

## Method

### Data

We use two main data sources: county-level population projections and gridded daily temperature projections. For *population aging*, we use current (2020) population counts and projections for midcentury (2050). Midcentury projections are based on data generated by Striessnig et al. (2019), who estimated decennial population size and composition by age group for 3,109 counties in the coterminous 48 United States over the period 2020–2100. County-level population projections (by age group and sex) are generated by simulating future demographic and migration dynamics under five shared socioeconomic pathways (SSPs) scenarios. The five SSPs (sustainability, middle-of-the-road, regional rivalry, inequality, fossil-fueled development) have been widely used in climate change research (Striessnig et al., 2019). We focus on projections of the share of the population aged 69 and older. Our threshold of age 69+ (rather than the widely used cut point of age 65+) is due to predefined age groups for which the population projections are provided (Meade et al., 2020).

*Temperature data* are from the NASA Earth Exchange Global Daily Downscaled Projections (NEX-GDDP-CMIP6) data set (Thrasher et al., 2022). We use daily average and maximum temperature outputs of 32 global climate models simulated under the Coupled Model Intercomparison Project Phase 6 (CMIP6; Eyring et al., 2016) ScenarioMIP exercise, bias-corrected, and downscaled to a global 0.25° grid. For consistency with our population data, gridded temperature series are averaged at the county level. Contemporary climate estimates are based on simulations of the 1995–2014 period, and midcentury (labeled “2050”) projections are based on simulations for the 2041–2060 period. For climate projections, we consider scenarios representing the fusion of the SSP scenarios and representative concentration pathway (RCP) scenarios, which reflect different use and emissions of air pollutants and greenhouse gasses (O’Neill et al., 2016).

Projections are based on two future scenarios widely used in climate research: SSP585 and SSP245 (Fricko et al., 2017; Kriegler et al., 2017). SSP585 represents a combination of the “fossil-fueled development” scenario (SSP5) and the high-warming scenario (RCP 8.5), whereas SSP245 represents the interplay of the “middle of the road” pathway of global socioeconomic development (SSP2) and moderate warming scenario (RCP 4.5). Given the inherent uncertainty of forecasting, we use two scenarios to provide insights into plausible alternative futures and their impacts on heat-related vulnerability. Empirical assessments of the likelihood of different climate scenarios conclude that there is “no clear consensus” on which pathway is most plausible (Huard et al., 2022). We focus primarily on results for the more extreme scenario (SSP585), which has been characterized as “relatively likely up until 2060” (Huard et al., 2022; 13) and highlight the modest differences detected using the more moderate projections (SSP245).

### Measures and Statistical Methods

We transform NASA NEX-GDDP-CMIP6 daily average and maximum temperature records to construct two meteorological indicators of individual heat exposure. *Cooling degree days* (*CDD*) measure chronic heat exposure over the course of a year and the 20-year *95th percentile of maximum daily temperatures* (*TMax*_*95*_) captures acute exposure to heat extremes.

CDDs are calculated as the excess of daily average temperatures over the threshold 75.2°F (24°C), cumulated over days in each year. This threshold temperature of 75.2°F is considered the minimum temperature at which air conditioning becomes necessary (Pavanello et al., 2021). Annual CDD values are largely determined by the duration of the hot season and the amplitude of peak temperatures. For example, a CDD of 1 refers to 1 day on which average diurnal temperature exceeds the 75.2° threshold by 1 degree. An average annual CDD of 450°F could reflect a climate with a long but moderate summer (e.g., 90 days with an average temperature of 82°F, 5°F over threshold) or a shorter but hotter summer (e.g., 60 days with an average temperature of 87.5°F, 7.5°F over the threshold). Turning to acute heat exposures, *TMax*_95_ is calculated based on each county’s within-time period distribution of simulated daily maximum temperatures. For example, a county where the 95th percentile of daily temperatures averaged over 20 years is 101°F is exposed to more severe heat risk than a county where the comparable 95th percentile temperature is 87°F.

We also construct *cumulative population-level measures* that capture heat exposure attributable both to climate change and population aging. Cumulative population-level *chronic* heat exposure is measured as *person-degree days* (*PDD*). This equals the average CDD level multiplied by the population over 69 years of age, in each county and time period. Cumulative population-level *acute* heat exposure is measured as person-degrees (*PD*). PD is the product of the extreme temperature threshold (TMax_95_) multiplied by the population over age 69 in each county and time period.

Finally, we estimate *sources of change* in population exposure over the first half of the 21st century. Using decomposition methods, we calculate the proportion change in older adults’ chronic heat exposure (PDD) that is attributable to each of the three theorized drivers: climate change, operationalized as ambient heat (CDD); population size, measured as the total population; and population aging, operationalized as the proportion of the population age 69+. We carry out decompositions at both the state and regional levels. Regions refer to the nine (coterminous) U.S. Census (2020a) divisions: New England, Middle Atlantic, East North Central, West North Central, South Atlantic, East South Central, West South Central, Mountain, and Pacific (see Table 1 for further description).

**Table 1.** Age 69+ Heat Exposure Determinants by U.S. Census Division, Based on Contemporary Indicators (1995–2014) and Midcentury (2050) Projections Using SSP585 and SSP245 Assumptions

	Population (millions)	Percentage age 69+	Pop. weighted CDDs (°F)	Pop. weighted TMax_95_ (°F)	PDD (million person degree days, °F)	PD_95_ (million person degrees, °F)
Contemporary						
United States	331.5	11.0	404	93	14,797	3,396
Northeast						
New England	16.6	12.2	84	87	170	177
Middle Atlantic	47.1	11.8	186	90	1,029	496
Midwest						
East North Central	54.0	11.7	153	90	990	567
West North Central	22.7	11.8	271	94	729	252
South						
South Atlantic	60.9	11.6	780	95	5,491	669
East South Central	19.8	11.3	497	95	1,109	213
West South Central	37.1	9.2	987	101	3,381	345
West						
Mountain	21.6	10.6	534	97	1,214	221
Pacific	51.8	9.8	131	89	665	453
Projected 2050 climate (SSP585)						
United States	469.7	16.6	700 (602, 788)	97 (96, 98)	54,674 (47,019, 61,555)	7,554 (7,516, 7,633)
Northeast						
New England	23.6	18.9	233 (155, 305)	92 (91, 93)	1,041 (690, 1,362)	408 (405, 413)
Middle Atlantic	66.9	16.7	449 (283, 488)	94 (93, 95)	5,027 (3,172, 5,469)	1,054 (1,039, 1,069)
*Midwest*						
East North Central	76.4	17.7	404 (258, 454)	94 (93, 96)	5,459 (3,491, 6,135)	1,273 (1,260, 1,301)
West North Central	31.9	17.6	574 (498, 727)	99 (98, 101)	3,224 (2,794, 4,083)	557 (549, 566)
South						
South Atlantic	86.0	16.9	1160 (1021, 1258)	98 (98, 99)	16,889 (14,866, 18,315)	1,428 (1,421, 1,436)
East South Central	27.7	16.8	867 (787, 1035)	99 (98, 99)	4,038 (3664, 4819)	460 (456, 462)
West South Central	52.5	14.5	1504 (1287, 1714)	104 (103, 106)	11,470 (9,815, 13,075)	796 (786, 805)
West						
Mountain	30.6	16.6	793 (723, 892)	102 (101, 103)	4,034 (3,682, 4,538)	518 (512, 523)
Pacific	74.2	15.4	247 (225, 284)	94 (93, 94)	2,820 (2,576, 3,244)	1,069 (1,061, 1,078)
Projected 2050 climate (SSP245)						
United States	397.3	17.0	615 (554, 634)	96 (95, 96)	41,609 (37,464, 42,875)	6,461 (6,406, 6,497)
Northeast						
New England	20.0	19.3	197 (122, 232)	90 (90, 91)	762 (471, 895)	349 (348, 353)
Middle Atlantic	57.0	17.1	336 (239, 425)	93 (92, 94)	3,276 (2327, 4143)	905 (899, 914)
Midwest						
East North Central	64.6	18.1	260.9 (212, 351)	93 (92, 94)	3,050 (2483, 4099)	1,089 (1,075, 1,100)
West North Central	26.6	17.9	514 (483, 657)	97 (96, 98)	2,442 (2,295, 3,126)	463 (459, 466)
South						
South Atlantic	72.6	17.4	1026 (925, 1176)	97 (97, 98)	12,922 (11,659, 14,810)	1,221 (1216, 1236)
East South Central	22.9	17.3	776 (646, 898)	98 (96, 99)	3,080 (2,561, 3,561)	388 (382, 391)
West South Central	44.3	14.9	1548 (1302, 1645)	103 (102, 104)	10,247 (8,620, 10,885)	684 (674, 691)
West						
Mountain	25.9	17.0	791 (759, 813)	100 (100, 100)	3,481 (3,339, 3,577)	440 (438, 441)
Pacific	63.5	15.7	230 (222, 267)	91.9 (91, 92)	2,289 (2,218, 2,618)	916 (910, 921)

*Notes*: CDD = cooling degree days, a measure of cumulative heat exposure over a year; TMax_95_ = 20-year 95th percentile of maximum daily temperatures that measures acute exposure to heat extremes; PDD = person degree days; PD_95_ = person degrees at the 95th diurnal temperature percentile. Median division values (and interquartile ranges) are shown for population-weighted CDD, TMax_95_, PDD, and PD_95_, across 32 global climate models for projected midcentury scenarios shown in parentheses. Contemporary refers to 2020 data for total population and age 69+ measures, and the 1995–2014 period for climate measures. United States refers to the 48 contiguous states. The nine Census divisions include New England (CT, ME, MA, NH, RI, VT); Middle Atlantic (NJ, NY, PA), East North Central (IL, IN, MI, WI), West North Central (IA, KS, MN, MS, NE, ND, SD), South Atlantic (DE, FL, GA, MD, NC, SC, VA, DC, WV), East South Central (AL, KY, MS, TN), West South Central (AR, LA, OK, TX), Mountain (AZ, CO, ID, MT, NV, NM, UT, WY), and Pacific (CA, OR, WA).

## Results

### Regional Descriptive Statistics

Descriptive statistics for the contemporary and two projected midcentury scenarios (SSP585 and SSP245) are presented in Table 1. We show descriptive statistics aggregated at the Census division level. (Supplementary Table 1 in Supplementary Material presents state-level descriptive statistics, and Supplementary Figures 1–3 display county-level patterns.)

#### Total population

Between 2020 and 2050, the total U.S. (coterminous) population is projected to increase by 20% (332–397 million) in the middle-of-the-road SSP245 scenario and 42% (332–470 million) in the high-growth SSP585 scenario. The total U.S. population is concentrated in the South Atlantic and East North Central regions, and to a lesser extent states in the Pacific and Mid-Atlantic. This geographic distribution persists at midcentury, in both the SSP245 and SSP585 scenarios.

#### Population aging

Our projections confirm that the U.S. population is aging significantly, with the fraction of population aged 69+ projected to increase from 11% today to around 17% in 2050. Older populations are currently concentrated in the Northeast, particularly New England, the Midwest, and the South Atlantic, especially Florida. The population age 69+ is projected to increase by 40%–60% across both SSP scenarios, with the largest increases in the West South Central, Mountain, and Pacific regions (see Supplementary Figure 1A–C).

#### Climate change indicators

Across regions and for both SSP scenarios, we see high and rising levels of chronic (CDD) and acute (TMax_95_) heat exposure, although the magnitude of these patterns varies geographically. The high (SSP585) and moderate (SSP245) warming scenarios show similar patterns although the magnitude of change is more modest in the latter scenario. The number of CDDs is projected to increase in all regions, rising by a projected 73% (from 404 to 700) in the high-warming scenario and 52% in the moderate scenario (see Supplementary Figure 2A–C). This overall increase is driven by dramatic increases in cooler regions that currently experience few CDDs. For example, under the SSP585 scenario, we see a 250% increase in the Northeast, yet more modest increases (50%–75%) in Southern states where the bulk of CDDs are currently concentrated.

Current TMax_95_ estimates are in the low to mid 90°F range across much of the country, with the exception of the slightly cooler New England (87°F) and Pacific (89°F) regions and the hotter West South Central (101°F). In contrast to the considerable projected increases in CDDs, projected increases in acute heat exposure are more modest and do not differ markedly across the two scenarios (see Supplementary Figure 3A–C). At the national level, TMax_95_ is projected to increase by just 4% (93–97°F) under SSP585, with similar increases projected across the nine regions (ranging from 3% in West South Central to 5.7% in New England) and under the SSP245 scenario. Although the distribution of annual temperatures is moving upward for all regions, very extreme temperatures are not increasing as dramatically.

#### Cumulative heat exposure metrics.

Cumulative population-level exposure to chronic (PDD) and acute (PD) heat exposures show similar geographic patterns, although with less dramatic increases projected for acute exposure. PDDs currently are concentrated in the South Atlantic and West South Central regions, dominated by Florida and Texas, respectively. This pattern persists to 2050, but its amplitude increases markedly. In the high-warming (SSP585) scenario, chronic exposure (PDD) more than triples nationwide, rising by a factor of six in New England, five in the Mid-Atlantic and East North Central regions, four in the Pacific and West North Central regions, and three elsewhere. Comparable yet slightly smaller increases are projected under the moderate (SSP245) scenario. Acute heat exposures (PD) follow the same general patterns as PDD, although the magnitude of increase is more modest. PD is projected to increase 24% nationwide, 10%–20% across the South, and 60%–70% in the cooler climates of the East North Central and Pacific regions, as well as New England.

### County-Level Analyses

**Figure 1.**
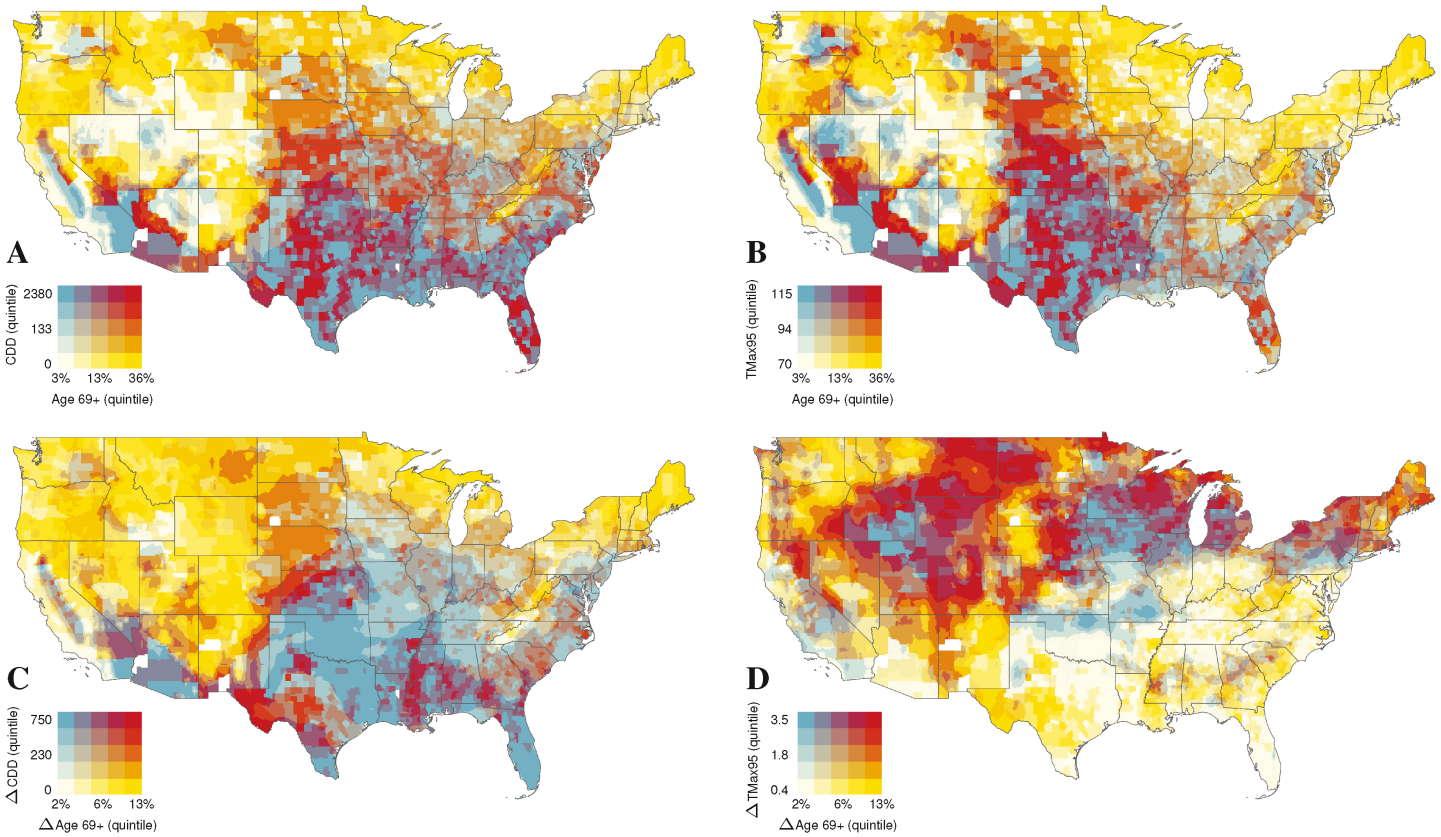
Percentage of population aged 69+: (A) annual cooling degree days, (B) 95th percentile of 20-year daily maximum temperatures, (C) contemporary indicators (1995–2014), and (D) projected change by 2050, based on SSP585 assumptions. *Notes*: CDD = cooling degree days, a measure of cumulative heat exposure over a year (left column); TMax_95_ = 20-year 95th percentile of maximum daily temperatures measures acute exposure to heat extremes (right side).

We present our county-level results in four maps visualizing the fraction of the population age 69+ and the average yearly CDD and TMax_95_ exposure for contemporary population counts (Figure 1A and B, respectively) and projected changes therein by 2050 (Figure 1C and D, respectively). We focus primarily on SSP585 for brevity, although similar patterns of slightly smaller magnitude are detected for SSP245. (Supplementary Figure 4A–D present maps for SSP245 patterns.)

For Figure 1A and B, the map key uses color-coding to depict a county’s share of older adults (X-axis) and CDDs or TMax_9*5*_ (Y-axis for Figure 1A and B, respectively). The key presents values in quintiles, to facilitate regional comparisons. Both maps show clear regional and county-level variation in the concentration of older adults and cumulative heat exposure. We focus on CDDs because similar patterns are found for TMax_95_, reflecting a 0.79 zero-order correlation for current values. The maps confirm high concentrations of older adults in the upper Midwest, upper New England, and parts of the Pacific Northwest and northern Mountain region (darker yellow), such that more than one-fifth of county residents are age 69+ Those regions currently have modest heat exposure, with CDD and TMax_95_ levels in the lowest quintile. Conversely, pockets of the Deep South, especially the coastal regions of Louisiana and Texas, and inland areas of southern California and southern Nevada evidence among the highest heat exposures yet very low proportions of older adults (deep blue).

The maps reveal “hotspots” with high concentrations of older adults and the highest levels of heat exposure (bright red). Most of Florida’s east coast (except Miami-Dade County), west coast, and north central regions are hotspots, as are some areas of central Texas, Oklahoma, and Arkansas. Low-risk areas (pale yellow) have small concentrations of both older adults and heat exposure. These areas are located primarily in rural regions of northern Nevada, Utah, and Wyoming.

Figure 1C and D show *change* in county-level population heat exposure under the SSP585 scenario. The map key refers to change (in quintiles) in CDD or TMax_95_ and population age structure over the contemporary through 2050 period. Results are remarkably similar for the SSP245 scenario (Supplementary Figure 4A–C). Figure 1C shows that high levels of population aging with more modest changes in CDDs will occur in largely rural areas. The dark yellow areas of Maine, northern and western New York, upper Michigan, Wisconsin, and northern parts of Minnesota, North Dakota, Montana, and the Pacific Northwest states, as well as rural areas in western parts of Wyoming, Colorado, and New Mexico and eastern Nevada reveal steep increases in share of the population ages 69+ with flatter increases in temperature exposure. Conversely, the flattest levels of population aging with the steepest increases in CDDs (bright blue) are concentrated in Florida, Oklahoma, the northern and eastern regions of Texas, western Louisiana, parts of Arkansas, desert regions of California, and southern Arizona.

Areas with the greatest increase in both risks, with increasing concentrations of older populations and CDDs (bright red) are clustered in the largely rural areas in southwest Texas, pockets of Kansas and Nebraska, coastal and bayou areas of Louisiana, and western Mississippi. Other at-risk areas include several major metropolitan areas that will experience substantial increases in temperature, although more modest population aging (medium blue and light orange areas). These areas include greater New York City, southern New England, Delaware, and greater Detroit.

Similar patterns are evidenced for TMax_95_ with a few notable exceptions. Figure 1D shows that the nation’s historically hottest counties will not see dramatic increases in their temperature extremes, as noted by the yellow tint throughout much of the south and southwest. However, pockets in Texas, Arizona, and the Deep South will see considerable increases in their age 69+ population. By contrast, historically colder areas of the Northeast and upper Midwest will see proportionally larger increases in exposure to temperature extremes.

Figure 1C and D show projected *change*, yet do not show *absolute levels* of projected population heat exposure. Areas revealing modest change in a particular dimension may still fare poorly with respect to the total *magnitude* of exposure. For example, Florida is projected to have a slight increase in the share of the population ages 69+. However, the state-level descriptive statistics presented in Supplementary Table 1 show that under both the SSP245 and SSP585 scenarios, Florida ranks second highest in the nation for *population-weighted* CDDs, with projected values of 1,807 and 1,714, respectively. By contrast, California—the nation’s current and projected most populous state—has comparable population-weighted CDDs of 306 and 290 under the extreme and moderate scenarios, respectively. These CDDs demonstrate the absolute demands on social institutions to address older adults’ heat exposure.

### Decomposition Results

**Figure 2.**
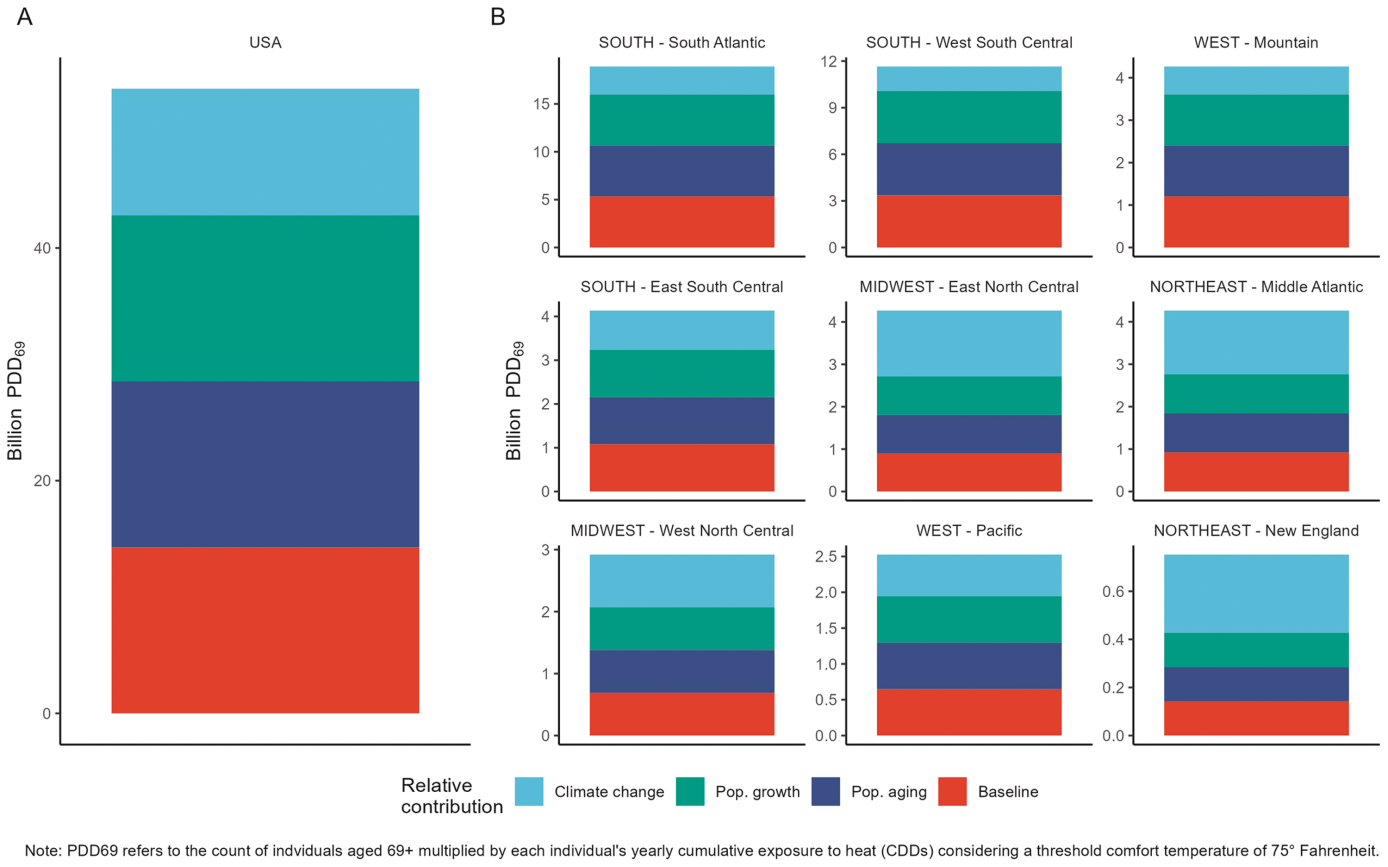
Relative contributions of climate change, population growth, and population aging to person degree days, by U.S. Census regions, 2050.

Figure 2 shows the relative share of billion PDDs attributable to population growth, population aging, and warming for the nine Census regions. (Supplementary Figure 5 shows state-level results.) PDD equals the total number of persons ages 69+ multiplied by each person’s yearly cumulative exposure to heat (CDDs), with a threshold comfort temperature of 75°F.

The United States overall reveals relatively equal contributions of the three drivers, with climate change playing a slightly smaller role than either population growth or population aging. The Census Division figures show variation, such that climate change is a larger driver of future exposure in historically colder regions, especially in New England and to a slightly lesser extent the Middle Atlantic, and Midwest. Climate change plays a lesser role, and overall population growth and population aging are larger drivers in three Southern regions and the Mountain region. The Pacific states of the West show relatively equal contributions of the three drivers. Supplementary Figure 5 shows further variation by state, even within the Census regions, such that climate change is the largest driver in the New England states ME, NH, and RI, Mountain states of WY and CO, and Midwest states of IA, MN, ND, and WI, and Western states of CO, MT, WY. Population aging is an especially substantial driver in southern and western states including AR, AZ, NV, and TX.

Although the relative impacts of climate change are most substantial in historically colder regions, it is important to also consider the *absolute magnitude* of these patterns. The Y-axes in Figure 2 show levels of overall exposure (PDD), revealing the greatest exposure in the South Atlantic region (which includes FL), with a projected PDD level of nearly 20 billion, followed by West South Central at around 12 billion. This is vastly larger than the total exposure of less than 1 billion in New England and less than 4 billion in the West and Midwest.

## Discussion

Our analysis is the first we know of to project the U.S. regions most vulnerable to older adults’ future chronic and acute heat exposure, and the drivers of these patterns, allowing us to outline implications for policy and practice (for comparable global analyses, see Park et al., 2020 and Tuholske et al., 2021). Our study yielded three key findings.

First, population aging and rising heat exposures are inevitabilities in the 21st century, although the current levels and magnitude of projected increases vary across and within U.S. regions. Our results broadly suggest a “tale of two countries,” whereby northern regions have relatively older populations with more modest heat exposure, and southern regions have relatively younger populations with more severe heat exposure. However, a cluster of regions stands out as “hotspots” distinguished both by high levels of heat exposure and older populations. These hotspots are concentrated in Florida’s coastal regions that are retirement destinations, and rural communities of the Southwest, especially in Texas and Oklahoma.

Second, historically warm southern regions will see significant increases in chronic heat exposure with flatter increases in their exposure to already-high heat extremes (i.e., TMax_95_). These regions also will experience the most dramatic increases in their share of the population ages 69+, a pattern we suspect is attributable to older adults’ desire to migrate to warmer climates. As a result, southern regions will face high demands for supports and services, as ever-growing concentrations of older adults require suitable housing, health care, and other infrastructures that enable them to adapt to heat exposure. A different scenario is predicted for historically colder areas of the Northeast, upper Midwest, and to a lesser extent the northwest, with a particular growth in extreme temperatures in the upper Northeast and Midwest. These areas will see flatter increases in population aging, but steady increases in chronic temperature exposure and particularly sharp increases in heat extremes. Rising heat exposure may pose substantial challenges to densely populated areas of the Northeast and Midwest with older housing stock and less efficient heating and cooling systems (U.S. Energy Information Administration, 2022).

Third, decomposition analyses revealed that climate change will account for a particularly large share of population heat exposure in historically colder locations, including the northeast regions of New England and the Middle Atlantic, and the Midwest regions of the East and West North Central. Conversely, population growth and population aging are larger drivers in three Southern regions (South Atlantic and both West and East South Central) and the Mountain region, consistent with the results from our county-level projections.

### Implications for Policy, Practice, and Theory

Our results suggest that different adaptations may be necessary in historically colder versus historically warmer areas, and these adaptations must address the distinctive needs of high and rising numbers of older adults. As the climate continues to warm, populations in northern states and at higher elevations who are not accustomed to heat waves may require education and early warning systems that provide advance notice of dangerous high temperatures. National Weather Service alerts could recommend protective measures such as using a fan or air conditioning, visiting a cooling center, avoiding overexertion, or increasing one’s water intake. Heat alerts also could instruct older adults to seek health care if needed, such as treatment for heat stroke.

Surprisingly, however, heat alerts have not been found to lower mortality risk (Weinberger et al., 2021). To ensure maximal effectiveness, messaging must be designed to educate older adults and encourage appropriate actions, especially in regions where residents may lack knowledge or minimize the importance of extreme heat events (Bedi et al., 2022). Residents of historically cooler U.S. regions perceive an unrealistically low risk of extreme heat threats, despite their intensifying exposures (Howe et al., 2019). More worrisome, older adults underestimate their personal vulnerability to heat extremes, despite their greater risk (Abrahamson et al., 2009). Gerontology and geriatrics practitioners’ expertise may be helpful in shaping the content and delivery of heat alert messaging. Home health workers also could be tasked with educating older adults about their heat vulnerability and providing educational materials and heating response checklists (Gamble et al., 2013).

Historically hot regions generally have the infrastructures, experience, and capacities to adapt to heat extremes. However, in many southern areas, population aging will put greater numbers of older adults at risk of heat exposure, intensifying strains on health care systems, public utilities, and other aspects of the infrastructure. These demands may overtax existing structures, such as public cooling centers, and will require adjustments to meet the distinctive needs of older adults. Older adults often lack knowledge about the location of cooling centers or have difficulty accessing these spaces due to insufficient transportation or physical mobility limitations (Bedi et al., 2022). Professionals serving older adults are well situated to provide information regarding cooling centers, encourage their use, and coordinate transport. Other community-level initiatives might include the development or expansion of registries and surveillance data on older adults, and the use of geographic information systems to help first responders identify those neighborhoods with the greatest concentrations of older adults (Davies & Bhutta, 2022).

Physical infrastructure adaptations, including the installation of air conditioning in institutional and residential settings, via energy efficiency incentives or building code mandates, also are critical. However, these interventions are costly and require additional electricity to provide sufficient cooling, especially during hot summer months in which demand for power peaks (Romitti & Sue Wing, 2022; Skarha et al 2021). As rising numbers of people of all ages, but especially older adults, rely on air conditioning to withstand high temperatures, this added burden on power systems can threaten their reliability, triggering blackouts precisely when the demand for electricity for cooling is largest. Costly investments in power generation, transmission, and distribution will be necessary, although such investments may pose challenges to under-resourced regions (Ralston Fonseca et al., 2021).

Infrastructure investments also may create negative unintended consequences for older adults. For example, in 2017, Florida mandated that long-term facilities install backup power systems capable of providing air conditioning for at least 4 days (Skarha et al., 2021). However, the high costs of these adaptations led to compliance delays, with some experts cautioning that these adaptations could divert funds from staffing and ultimately compromise the quality of care at some facilities (June et al., 2023). This case reveals the importance of climate-related policies that explicitly consider the simultaneous impacts of population aging.

Our study results have implications for theory and practice. Cumulative dis/advantage models have been particularly influential for understanding disparities in late-life well-being, with much of this work emphasizing individual-level factors such as education or parental economic resources as factors that may have multiplicative impacts on late-life well-being (Hatch, 2005). However, our work suggests that (dis)advantage also can accumulate at the population level, such that the co-occurrence of rising temperatures and growing aged populations will pose particular threats to older adults residing in these regions. The threats are further amplified in regions that lack sufficient economic resources to make the necessary adaptations to their public infrastructures or services.

### Future Directions

Our study is the first step of what we hope is a robust research program focused on the intersections of population aging and increasing heat exposures. However, this analysis has limitations that could be addressed in future work. First, we used a coarse marker of old age (age 69+) due to data constraints; future studies could consider more fine-grained age groups, especially oldest-old persons (ages 85+) who are particularly vulnerable to environmental threats. The State of Florida (2022) projects that their age 85+ population will experience the steepest growth of any age group during the period 2040–2050. “Hot spot” regions with high levels of heat exposure and rapidly growing oldest-old populations will require particularly heavy investments in their health care facilities, as care needs increase with age (Federal Interagency Forum on Aging-Related Statistics, 2020).

Future studies also could focus on additional sources of area-level heterogeneity, such as the share of low-income persons, urban versus rural populations, and solo-dweller households. For example, “heat island” effects may be observed in urban areas experiencing high temperatures, due to structures like roads and buildings that absorb heat more than natural terrains such as parks and forests (Lowe, 2016). Urban areas also are home to disproportionately large shares of older, lower-income, less educated, and racially minoritized populations—populations who are more likely to live in substandard housing with insufficient cooling systems (Romitti & Sue Wing, 2022). Areas with high concentrations of solo-dwelling older adults may face challenges with evacuation in the case of a heat emergency (Bascetta, 2006).

Population aging is inevitable given historical fertility and mortality trends in the United States, and rising heat levels are likely given current and projected emissions trajectories in the United States. We hope that our documentation of these co-occurring patterns starts a broader conversation about the impacts of climate change for regions with high and growing numbers of older adults. Such conversations can help set the foundation for devising age-sensitive climate change adaptations to ensure the well-being of older adults.

## Supplementary Material

gnad050_suppl_Supplementary_Material

## Data Availability

The data and materials used for our analyses are available to other researchers for replication purposes. The materials can be obtained by contacting the study authors. The studies reported in the manuscript are not pre-registered.
