# Supplementary material for: Population Aging and Heat Exposure in the 21st Century: Which U.S. Regions Are at Greatest Risk and Why?
Source: Gerontologist. 2023 Apr 28;64(3):gnad050. doi: 10.1093/geront/gnad050 (PMC10860513; doi:10.1093/geront/gnad050)
Supplement: gnad050_suppl_Supplementary_Material [file gnad050_suppl_supplementary_material.docx]

**Online Supplementary Material**

| Supplementary Table 1. Age 69+ Heat Exposure Determinants by U.S. State, Based on Contemporary Indicators (1995-2015) and Mid-Century (2050) Projections Using SSP585 and SSP245 Assumptions |  |
| --- | --- |
| Contemporary (1995-2014) |  |

|  | Population  (millions) | Percentage  age 69+ | Pop. weighted  CDDs (°F) | Pop. weighted  TMAX95 (°F) | PDD (Million  person degree days, °F) | PD95 (Million  person degrees, °F) |  |
| --- | --- | --- | --- | --- | --- | --- | --- |

| AL | 5.2 | 11.6 | 610.1 | 95.8 | 370.2 | 58.1 |  |
| --- | --- | --- | --- | --- | --- | --- | --- |
| AR | 3.1 | 11.8 | 673.3 | 98.9 | 247.3 | 36.3 |  |
| AZ | 6.2 | 11.8 | 1370.1 | 106.8 | 998.8 | 77.9 |  |
| CA | 40.6 | 9.4 | 166.7 | 90.1 | 635 | 343.2 |  |
| CO | 5.4 | 9.9 | 43.8 | 89.7 | 23.2 | 47.4 |  |
| CT | 3.9 | 12 | 105.7 | 88.1 | 50 | 41.7 |  |
| DE | 0.9 | 11.8 | 362.1 | 93.5 | 38 | 9.8 |  |
| FL | 18.8 | 14 | 1316.3 | 96.2 | 3471.3 | 253.8 |  |
| GA | 9.7 | 8.9 | 549.6 | 95 | 477.1 | 82.5 |  |
| IA | 3.4 | 12.9 | 162.8 | 91.3 | 72.5 | 40.7 |  |
| ID | 1.5 | 10.8 | 93.6 | 93.7 | 15.4 | 15.4 |  |
| IL | 14.6 | 10.7 | 187.7 | 90.8 | 291.8 | 141.2 |  |
| IN | 7 | 11 | 217.2 | 91.5 | 168.8 | 71.2 |  |
| KS | 3.2 | 11.2 | 516 | 98.8 | 186.8 | 35.8 |  |
| KY | 4.6 | 11.3 | 347.4 | 94 | 181.1 | 49 |  |
| LA | 5.2 | 10.5 | 966.9 | 97.5 | 528 | 53.2 |  |
| MA | 7.8 | 11.7 | 94 | 87.7 | 84.9 | 79.3 |  |
| MD | 6.7 | 10.3 | 378.4 | 94 | 261 | 64.8 |  |
| ME | 1.5 | 14.5 | 26.6 | 84.5 | 5.8 | 18.6 |  |
| MI | 12.1 | 12.4 | 111.2 | 88.8 | 166.4 | 132.8 |  |
| MN | 5.6 | 11.5 | 82.1 | 89.1 | 53.1 | 57.6 |  |
| MO | 6.6 | 11.7 | 418.5 | 95.8 | 321.5 | 73.6 |  |
| MS | 3.3 | 10.8 | 745.1 | 97.7 | 265.3 | 34.8 |  |
| MT | 1 | 14.1 | 26.7 | 89.6 | 3.8 | 12.7 |  |
| NC | 9.4 | 10.9 | 448.6 | 94.1 | 460.8 | 96.6 |  |
| ND | 0.7 | 13.4 | 55 | 90.8 | 5.3 | 8.7 |  |
| NE | 2.2 | 11.6 | 253.3 | 95.8 | 65.2 | 24.7 |  |
| NH | 1.5 | 12.2 | 61.6 | 87 | 11 | 15.6 |  |
| NJ | 10.1 | 10.9 | 235.3 | 90.9 | 260.3 | 100.6 |  |
| NM | 2 | 12 | 120.8 | 93.5 | 28.6 | 22.2 |  |
| NV | 2.4 | 9.4 | 563.9 | 101 | 125.7 | 22.5 |  |
| NY | 22.4 | 11.2 | 155.3 | 88.5 | 390.6 | 222.6 |  |
| OH | 14 | 12.3 | 176.6 | 90.7 | 305.3 | 156.8 |  |
| OK | 4 | 10.9 | 788.2 | 102 | 345.5 | 44.7 |  |
| OR | 4 | 12.4 | 14.9 | 87.1 | 7.5 | 43.6 |  |
| PA | 14.6 | 13.1 | 196.9 | 90.6 | 376.1 | 173 |  |
| RI | 1.2 | 12.5 | 87.6 | 86.6 | 13.7 | 13.5 |  |
| SC | 4.8 | 11.5 | 615.1 | 95.3 | 334.8 | 51.9 |  |
| SD | 0.9 | 12.4 | 151.7 | 94.4 | 16.8 | 10.5 |  |
| TN | 6.7 | 11.2 | 396 | 94.3 | 296.4 | 70.6 |  |
| TX | 24.8 | 8.4 | 1074.9 | 101.5 | 2229.9 | 210.6 |  |
| UT | 2.6 | 7.3 | 82.9 | 91.4 | 15.5 | 17.1 |  |
| VA | 8.4 | 10.3 | 397.9 | 93.8 | 342.9 | 80.8 |  |
| VT | 0.7 | 13.9 | 42.8 | 85.5 | 4.2 | 8.3 |  |
| WA | 7.2 | 10.7 | 29.8 | 85.7 | 22.9 | 66 |  |
| WI | 6.3 | 11.8 | 69.1 | 87.6 | 50.9 | 64.6 |  |
| WV | 2.1 | 14.3 | 197.8 | 91.2 | 60.7 | 28 |  |
| WY | 0.6 | 11.4 | 20.2 | 89.4 | 1.3 | 5.8 |  |
| USA | 331.5 | 11 | 404.3 | 92.8 | 14796.8 | 3396.2 |  |
|  |  |  |  |  |  |  |  |

|  | Projected 2050 climate (SSP 585) | | | | | |  |
| --- | --- | --- | --- | --- | --- | --- | --- |
|  | Population  (millions) | Percentage  age 69+ | Pop. weighted  CDDs (°F) | Pop. weighted  TMAX95 (°F) | PDD (Million  person degree days, °F) | PD95 (Million  person degrees, °F) |  |
| AL | 7.3 | 17.1 | 1040.7  (954.5, 1154.4) | 98.5  (97.9, 99.5) | 1300.1  (1192.5, 1442.2) | 123  (122.3, 124.3) |  |
| AR | 4.3 | 17 | 1098.5  (970.6, 1428.3) | 103.1  (101.6, 103.3) | 800.8  (707.6, 1041.2) | 75.1  (74.1, 75.3) |  |
| AZ | 8.9 | 17.3 | 2000.6  (1891.4, 2178.1) | 111.3  (110.2, 111.7) | 3079  (2911, 3352.2) | 171.3  (169.5, 172) |  |
| CA | 58.3 | 14.8 | 305.7  (269.9, 349.4) | 94.2  (93.7, 95.3) | 2630.5  (2321.9, 3006.6) | 810.6  (805.9, 819.7) |  |
| CO | 7.6 | 16.4 | 126.3  (71, 202) | 94.4  (93.6, 96.4) | 158  (88.9, 252.7) | 118.1  (117, 120.6) |  |
| CT | 5.6 | 18.6 | 307.3  (191, 380.7) | 92.2  (91.5, 93.1) | 321.9  (200, 398.7) | 96.6  (95.8, 97.5) |  |
| DE | 1.3 | 17.1 | 728  (579.1, 801.2) | 97.2  (96.2, 98.7) | 158.4  (126, 174.4) | 21.1  (20.9, 21.5) |  |
| FL | 26.7 | 18.7 | 1807.5  (1671.3, 1962.1) | 99.1  (98.5, 99.7) | 8998  (8320.4, 9767.7) | 493.5  (490.5, 496.4) |  |
| GA | 13.8 | 14.6 | 930.1  (870.7, 1039.6) | 97.9  (97.6, 98.8) | 1873.2  (1753.5, 2093.6) | 197.2  (196.5, 199) |  |
| IA | 4.8 | 18.5 | 456.8  (363.6, 543.6) | 97.1  (95.3, 99) | 405.8  (323.1, 483) | 86.3  (84.7, 87.9) |  |
| ID | 2.1 | 16.9 | 234.2  (196.7, 368.7) | 99.5  (98.3, 100.2) | 84.4  (70.9, 132.8) | 35.8  (35.4, 36.1) |  |
| IL | 20.8 | 16.3 | 472.8  (334.3, 532.9) | 95.3  (93.9, 97.3) | 1600.4  (1131.7, 1803.7) | 322.4  (318, 329.2) |  |
| IN | 10 | 16.6 | 561.4  (359.7, 586.6) | 95.7  (94.4, 97.8) | 929  (595.2, 970.7) | 158.3  (156.2, 161.9) |  |
| KS | 4.6 | 16.9 | 940.8  (692.4, 1192.1) | 104.4  (103.1, 105.3) | 727.2  (535.2, 921.5) | 80.7  (79.7, 81.4) |  |
| KY | 6.4 | 17 | 679.6  (519.5, 831.1) | 97.7  (96.8, 99.7) | 740  (565.8, 905) | 106.4  (105.5, 108.6) |  |
| LA | 7.3 | 16.3 | 1413  (1324.8, 1565.9) | 100.7  (98.9, 101.3) | 1675.9  (1571.2, 1857.2) | 119.4  (117.3, 120.1) |  |
| MA | 11.1 | 18.2 | 253.8  (174.3, 338.2) | 92.1  (91.1, 92.9) | 513  (352.1, 683.5) | 186.1  (184.1, 187.7) |  |
| MD | 9.6 | 15.8 | 747.9  (573.1, 778.2) | 97.9  (97, 99.8) | 1134.8  (869.5, 1180.6) | 148.5  (147.2, 151.5) |  |
| ME | 2.1 | 22.2 | 74.2  (50.3, 121.1) | 89.1  (87.7, 90.3) | 34.2  (23.2, 55.8) | 41.1  (40.4, 41.6) |  |
| MI | 17.1 | 19 | 309.1  (185.7, 370.9) | 93.1  (92.2, 95) | 1004  (603, 1204.6) | 302.5  (299.5, 308.6) |  |
| MN | 7.8 | 17.7 | 243.8  (187.2, 380.6) | 95.6  (93.6, 96.9) | 338.4  (259.8, 528.2) | 132.7  (129.9, 134.5) |  |
| MO | 9.3 | 16.9 | 785.8  (700.2, 1019.8) | 101.4  (99.3, 102) | 1233  (1098.6, 1600.2) | 159.2  (155.8, 160) |  |
| MS | 4.6 | 16.8 | 1182.7  (1065.8, 1391.5) | 100.4  (100.1, 101.9) | 902.2  (813.1, 1061.5) | 76.6  (76.3, 77.7) |  |
| MT | 1.4 | 21 | 76.3  (55.4, 202.8) | 95.2  (94.2, 97) | 22.2  (16.1, 58.9) | 27.6  (27.4, 28.2) |  |
| NC | 13.3 | 16.5 | 738.2  (675.6, 901.8) | 97.2  (96.4, 97.5) | 1611.2  (1474.6, 1968.1) | 212.2  (210.5, 212.8) |  |
| ND | 1 | 20.4 | 195  (155.7, 399.2) | 97.9  (95.8, 99) | 39.4  (31.5, 80.7) | 19.8  (19.4, 20) |  |
| NE | 3.2 | 17.7 | 601.1  (479.8, 694.8) | 101.3  (99.5, 103.3) | 336.6  (268.6, 389) | 56.7  (55.7, 57.8) |  |
| NH | 2 | 19.2 | 152.1  (101.4, 197.6) | 91.6  (90.5, 92.7) | 59.8  (39.8, 77.7) | 36  (35.6, 36.4) |  |
| NJ | 14.4 | 16 | 575.2  (382.8, 600.2) | 95.1  (94, 96.3) | 1324.5  (881.3, 1382) | 218.9  (216.6, 221.7) |  |
| NM | 2.8 | 18.5 | 260.2  (223.3, 308.4) | 98.4  (97, 99.3) | 134.1  (115.1, 159) | 50.7  (50, 51.2) |  |
| NV | 3.4 | 15.1 | 902.3  (823.2, 1015.7) | 106.2  (104.8, 107.2) | 463.3  (422.7, 521.6) | 54.5  (53.8, 55.1) |  |
| NY | 31.8 | 15.8 | 390.7  (246.1, 434.2) | 92.7  (91.8, 93.9) | 1957.9  (1233.4, 2176.2) | 464.5  (460, 470.8) |  |
| OH | 19.9 | 18.3 | 425.4  (265, 467) | 94.6  (93.8, 96.9) | 1549.4  (965.3, 1701) | 344.7  (341.5, 353) |  |
| OK | 5.6 | 15.3 | 1236.1  (1045.5, 1637.6) | 106.4  (104.8, 107.5) | 1062.9  (899, 1408) | 91.5  (90.1, 92.4) |  |
| OR | 5.7 | 18.9 | 54  (42.5, 74.2) | 91.4  (90.9, 92) | 58.1  (45.7, 79.8) | 98.3  (97.7, 99) |  |
| PA | 20.7 | 18.8 | 429.7  (276.2, 475.1) | 95.2  (93.7, 96.7) | 1669.8  (1073.3, 1846.3) | 370.1  (364.1, 375.9) |  |
| RI | 1.8 | 19.5 | 261.5  (181.8, 345.9) | 90.6  (90, 91.4) | 91  (63.2, 120.4) | 31.5  (31.3, 31.8) |  |
| SC | 6.7 | 17.4 | 978.1  (910.5, 1139.7) | 98.6  (97.8, 99) | 1136.2  (1057.6, 1323.8) | 114.5  (113.6, 115) |  |
| SD | 1.2 | 18.8 | 337.1  (273.8, 530.4) | 101.2  (98.4, 102.4) | 78.2  (63.5, 123) | 23.5  (22.8, 23.7) |  |
| TN | 9.4 | 16.5 | 753.2  (680.8, 925.8) | 97.8  (97, 98.6) | 1171  (1058.5, 1439.3) | 152  (150.7, 153.3) |  |
| TX | 35.3 | 13.7 | 1543.5  (1389.5, 1802.1) | 105.2  (103.9, 106.6) | 7489.3  (6742.1, 8743.9) | 510.4  (504.2, 517.1) |  |
| UT | 3.6 | 13.2 | 217.9  (149, 255.9) | 96.5  (95, 97.2) | 103.8  (71, 121.9) | 46  (45.3, 46.3) |  |
| VA | 11.9 | 15.8 | 729.4  (625.9, 823.8) | 97  (96.4, 98.8) | 1373.3  (1178.5, 1551) | 182.6  (181.4, 185.9) |  |
| VT | 0.9 | 20.5 | 105.4  (58.4, 133.4) | 89.9  (89.4, 91) | 20.4  (11.3, 25.9) | 17.4  (17.3, 17.6) |  |
| WA | 10.2 | 17.2 | 90.5  (65.5, 105.4) | 90.5  (89.7, 91.2) | 158.3  (114.5, 184.3) | 158.1  (156.9, 159.4) |  |
| WI | 8.7 | 18.2 | 207.8  (172.6, 299.7) | 93.1  (91.2, 93.7) | 328.4  (272.8, 473.6) | 147.1  (144.1, 148) |  |

| WV | 2.9 | 20.5 | 408.7  (290.4, 514.1) | 94.8  (94.2, 97.6) | 246  (174.8, 309.4) | 57  (56.7, 58.7) |  |
| --- | --- | --- | --- | --- | --- | --- | --- |
| WY | 0.8 | 18.1 | 72.9  (38.2, 120.1) | 94.9  (93.6, 96.3) | 10.5  (5.5, 17.3) | 13.6  (13.5, 13.9) |  |
| USA | 469.7 | 16.6 | 699.7  (601.7, 787.7) | 96.7  (96.2, 97.7) | 54674  (47019.3, 61554.6) | 7553.8  (7516, 7632.6) |  |
|  | Projected 2050 climate (SSP 245) | | | | | |  |
|  | Population  (millions) | Percentage  age 69+ | Pop. weighted  CDDs (°F) | Pop. weighted  TMAX95 (°F) | PDD (Million  person degree days, °F) | PD95 (Million  person degrees, °F) |  |
| AL | 6 | 17.6 | 920.5  (764.5, 1089.5) | 98  (96.6, 99.1) | 977.5  (811.8, 1156.9) | 104  (102.6, 105.2) |  |
| AR | 3.5 | 17.4 | 1079.7  (928.2, 1180) | 101.6  (99.8, 102.1) | 662.3  (569.3, 723.8) | 62.3  (61.2, 62.6) |  |
| AZ | 7.5 | 17.7 | 1948.6  (1853.7, 2021.7) | 109.8  (109.1, 110.1) | 2608.9  (2481.9, 2706.9) | 147.1  (146.1, 147.4) |  |
| CA | 50 | 15.1 | 290.1  (269.1, 313.3) | 92.9  (91.8, 93.4) | 2187.3  (2029.1, 2362) | 700.8  (692.5, 704.4) |  |
| CO | 6.5 | 16.8 | 109.6  (73.4, 167.9) | 93.1  (92.3, 93.6) | 119.4  (80, 182.9) | 101.4  (100.5, 101.9) |  |
| CT | 4.8 | 19.1 | 256.1  (136.5, 286.7) | 91  (90.7, 92.3) | 234.2  (124.9, 262.2) | 83.2  (82.9, 84.4) |  |
| DE | 1.1 | 17.5 | 580.6  (477.4, 680.8) | 96.2  (95.7, 97.5) | 108.9  (89.5, 127.7) | 18.1  (18, 18.3) |  |
| FL | 22.9 | 19.2 | 1714.2  (1607.6, 1958.3) | 97.9  (96.8, 98.2) | 7525.5  (7057.8, 8597.5) | 430  (425.1, 431.3) |  |
| GA | 11.5 | 15 | 809.9  (699.3, 996.1) | 97  (96.1, 97.8) | 1397.3  (1206.5, 1718.6) | 167.4  (165.8, 168.7) |  |
| IA | 4 | 18.7 | 337.4  (283.8, 467.7) | 95  (93.8, 96) | 251.5  (211.6, 348.7) | 70.8  (69.9, 71.6) |  |
| ID | 1.8 | 17.3 | 250.7  (184.2, 283.2) | 97.1  (96, 97.5) | 77.4  (56.9, 87.5) | 30  (29.7, 30.1) |  |
| IL | 17.7 | 16.6 | 301.9  (264.2, 441.1) | 94  (92.8, 94.9) | 886.4  (775.8, 1295.2) | 276.1  (272.5, 278.8) |  |
| IN | 8.3 | 17 | 349  (289.9, 463.6) | 94.5  (93.3, 95.9) | 495.7  (411.8, 658.6) | 134.2  (132.5, 136.3) |  |
| KS | 3.8 | 17.1 | 944.4  (880.6, 1075.4) | 102.8  (101.4, 103.9) | 614.2  (572.8, 699.5) | 66.9  (66, 67.6) |  |
| KY | 5.3 | 17.5 | 521.6  (450.7, 635.9) | 96.6  (95.5, 98.9) | 481.7  (416.2, 587.2) | 89.2  (88.2, 91.3) |  |
| LA | 6.1 | 16.8 | 1439.4  (1237.7, 1591.5) | 100.3  (98.8, 100.9) | 1484.3  (1276.3, 1641.1) | 103.4  (101.9, 104) |  |
| MA | 9.5 | 18.7 | 216  (139.8, 253.8) | 90.9  (90.5, 92) | 383.7  (248.2, 450.7) | 161.4  (160.7, 163.4) |  |
| MD | 8.2 | 16.2 | 559.4  (483.8, 656.2) | 96.9  (96, 98.5) | 740.6  (640.5, 868.7) | 128.3  (127.1, 130.4) |  |
| ME | 1.7 | 22.3 | 62.3  (36.7, 95.9) | 88  (86.6, 88.3) | 23.6  (13.9, 36.4) | 33.4  (32.9, 33.5) |  |
| MI | 14.4 | 19.6 | 224.5  (143.4, 278) | 91.9  (91, 92.5) | 631.9  (403.8, 782.5) | 258.6  (256.2, 260.5) |  |
| MN | 6.6 | 17.9 | 223.3  (134.7, 263.3) | 92.5  (91.8, 93.4) | 264  (159.3, 311.3) | 109.4  (108.6, 110.5) |  |
| MO | 7.7 | 17.4 | 740.5  (662.7, 863.1) | 99.6  (98, 100.7) | 996.9  (892.2, 1161.9) | 134.1  (131.9, 135.5) |  |
| MS | 3.7 | 17.3 | 1134.3  (922.7, 1258.2) | 100.7  (99, 101.5) | 731  (594.6, 810.8) | 64.9  (63.8, 65.4) |  |
| MT | 1.1 | 21.2 | 108.9  (61.3, 122.4) | 92.7  (91.6, 93.4) | 26.3  (14.8, 29.5) | 22.4  (22.1, 22.5) |  |
| NC | 11 | 16.9 | 628.1  (538, 750.8) | 96.3  (95.5, 97) | 1166.5  (999.2, 1394.4) | 178.8  (177.3, 180.2) |  |
| ND | 0.8 | 20.5 | 150.6  (117.7, 177.3) | 94.4  (93.7, 95.6) | 25.3  (19.8, 29.8) | 15.8  (15.7, 16) |  |
| NE | 2.6 | 17.9 | 583.5  (463.2, 692.6) | 99.1  (98.3, 99.5) | 275.3  (218.6, 326.8) | 46.8  (46.4, 47) |  |
| NH | 1.7 | 19.4 | 128.6  (74.2, 150.9) | 90.4  (89.8, 90.7) | 42.3  (24.4, 49.6) | 29.7  (29.5, 29.8) |  |
| NJ | 12.4 | 16.4 | 434.5  (318.7, 505.6) | 93.8  (93.4, 95) | 883.1  (647.6, 1027.6) | 190.6  (189.8, 193.1) |  |
| NM | 2.3 | 18.9 | 228.4  (180.3, 261.6) | 96.4  (95.6, 97.2) | 100.6  (79.4, 115.2) | 42.4  (42.1, 42.8) |  |
| NV | 2.9 | 15.3 | 932.6  (828.9, 970.6) | 103.8  (103.3, 104.6) | 411.5  (365.8, 428.3) | 45.8  (45.6, 46.1) |  |
| NY | 27.2 | 16.1 | 310.2  (197.9, 371.9) | 91.5  (90.9, 92.4) | 1357.2  (865.8, 1626.8) | 400.2  (397.5, 404.3) |  |
| OH | 16.9 | 18.8 | 264.1  (220.2, 350.8) | 93.7  (92.7, 94.8) | 835.9  (697, 1110.3) | 296.5  (293.3, 300) |  |
| OK | 4.7 | 15.8 | 1279.8  (1174.5, 1507.8) | 104.6  (103.4, 106.5) | 942.3  (864.7, 1110.1) | 77  (76.1, 78.4) |  |
| OR | 4.8 | 19.2 | 45.1  (21.4, 64.3) | 89.6  (88.8, 90.2) | 41.6  (19.8, 59.4) | 82.8  (82.1, 83.3) |  |
| PA | 17.4 | 19.2 | 321.8  (243.2, 434.8) | 93.8  (92.9, 94.6) | 1076.8  (813.9, 1454.9) | 313.7  (310.8, 316.7) |  |
| RI | 1.5 | 20 | 213.9  (136.4, 261.9) | 89.7  (89.3, 90.7) | 65.2  (41.6, 79.9) | 27.4  (27.2, 27.7) |  |
| SC | 5.6 | 17.9 | 863.4  (766.4, 1049.3) | 97.4  (96.8, 98) | 858.3  (761.9, 1043.2) | 96.8  (96.3, 97.4) |  |
| SD | 1 | 18.9 | 359.8  (275.8, 420.5) | 97.4  (96.9, 98.3) | 68.9  (52.8, 80.5) | 18.6  (18.5, 18.8) |  |
| TN | 7.9 | 17 | 648.5  (537.5, 740.4) | 96.9  (95.4, 97.8) | 867.3  (718.9, 990.1) | 129.6  (127.6, 130.8) |  |
| TX | 30 | 14.1 | 1687.7  (1410.4, 1783.9) | 104  (102.9, 105) | 7152.7  (5977.2, 7560.3) | 440.6  (435.9, 445.1) |  |
| UT | 3.1 | 13.7 | 203.8  (161, 249.7) | 94.3  (93.1, 94.6) | 85.9  (67.9, 105.3) | 39.8  (39.2, 39.9) |  |
| VA | 10 | 16.2 | 588.3  (501.2, 634.1) | 96.4  (95.5, 97.8) | 950.6  (809.9, 1024.6) | 155.7  (154.3, 158.1) |  |
| VT | 0.8 | 20.6 | 73.6  (36.9, 97.6) | 88.8  (88, 89) | 11.6  (5.8, 15.4) | 14  (13.9, 14) |  |
| WA | 8.7 | 17.4 | 87  (36.3, 110.9) | 88.5  (87.4, 89.4) | 131.1  (54.7, 167.1) | 133.3  (131.7, 134.6) |  |
| WI | 7.3 | 18.6 | 175.4  (97.1, 248.8) | 90.4  (90, 91.7) | 237.1  (131.3, 336.4) | 122.2  (121.7, 124) |  |
| WV | 2.4 | 21.1 | 279.2  (252.2, 384.5) | 94.1  (92.9, 95.9) | 141  (127.4, 194.1) | 47.5  (46.9, 48.4) |  |
| WY | 0.7 | 18.3 | 70.5  (50.6, 109.7) | 92.4  (91.9, 93.3) | 8.5  (6.1, 13.2) | 11.1  (11, 11.2) |  |
| USA | 397.3 | 17 | 615.4  (554.1, 634.1) | 95.6  (94.7, 96.1) | 41608.6  (37464, 42875.4) | 6460.9  (6405.5, 6497.1) |  |
|  |  |  |  |  |  |  |  |
| *Notes:* Contemporary refers to 2020 data for total population and age 69+ measures, and the 1995-2014 period for climate measures.  CDD: cooling degree days, a measure of cumulative heat exposure over a year; TMax95: 20-year 95th percentile of maximum daily temperatures (T95) that measures acute exposure to heat extremes; PDD: person degree days; PD95: person degrees at the 95th diurnal temperature percentile. Median values are presented for population weighted CDDs, TMX95, PDDs, and PD95, with interquartile ranges (in parentheses) for projected mid-century (2050) scenarios. | | | | | | |  |

Supplementary Figure 1A-C. Proportion of Population Age 69+ in 2020 and Projections for 2050, Based on SSP245 and SSP585 Projections.


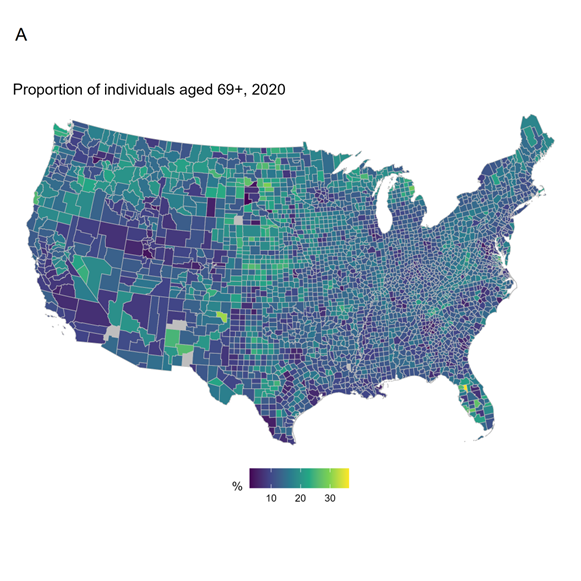

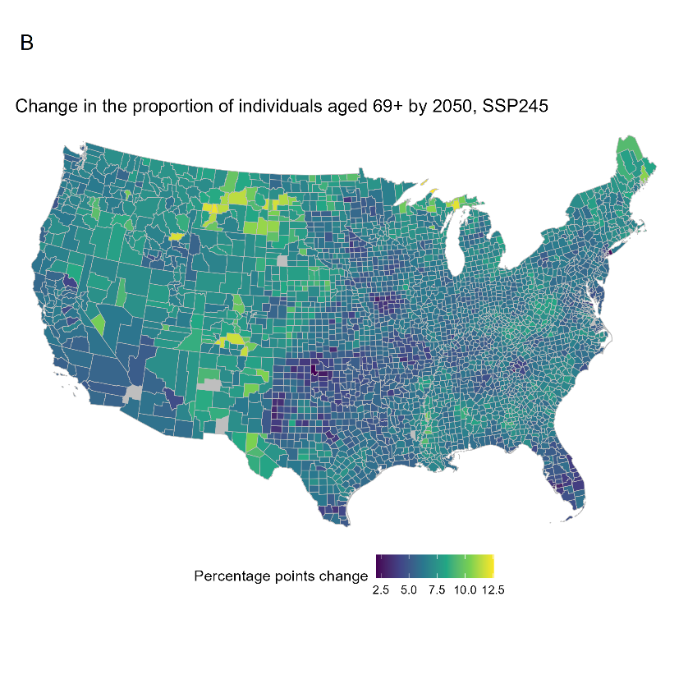


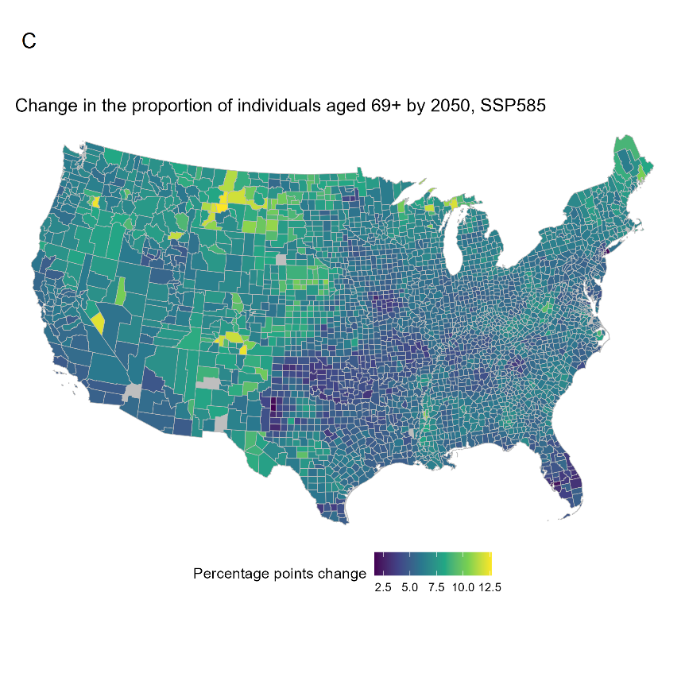


Supplementary Figures 2A to 2C. Annual Cooling Degree Days (CDDs) in Early 21st Century (1995-2014) and Projected Change by 2050, Based on SSP245 and SSP585 Assumptions


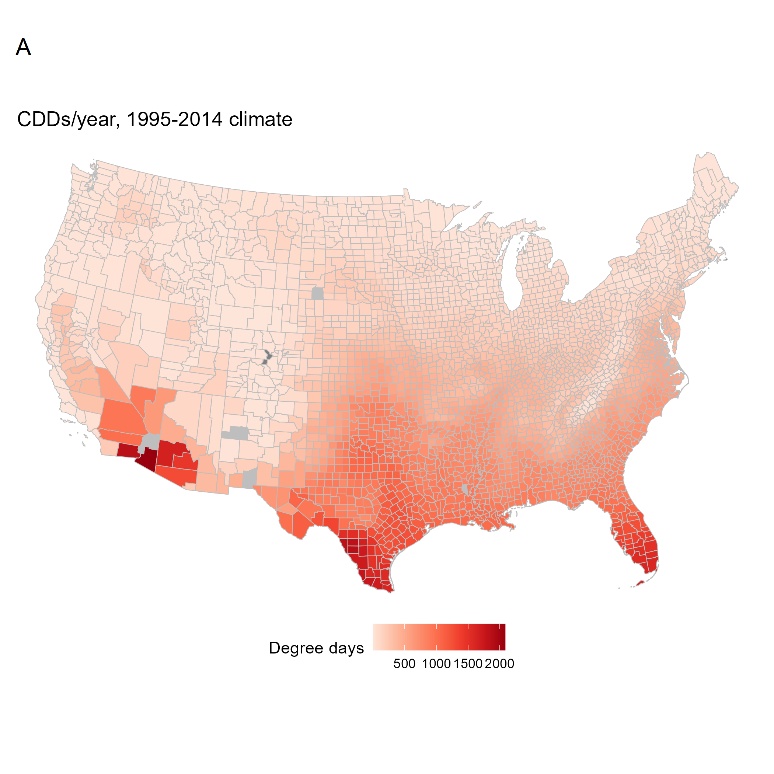

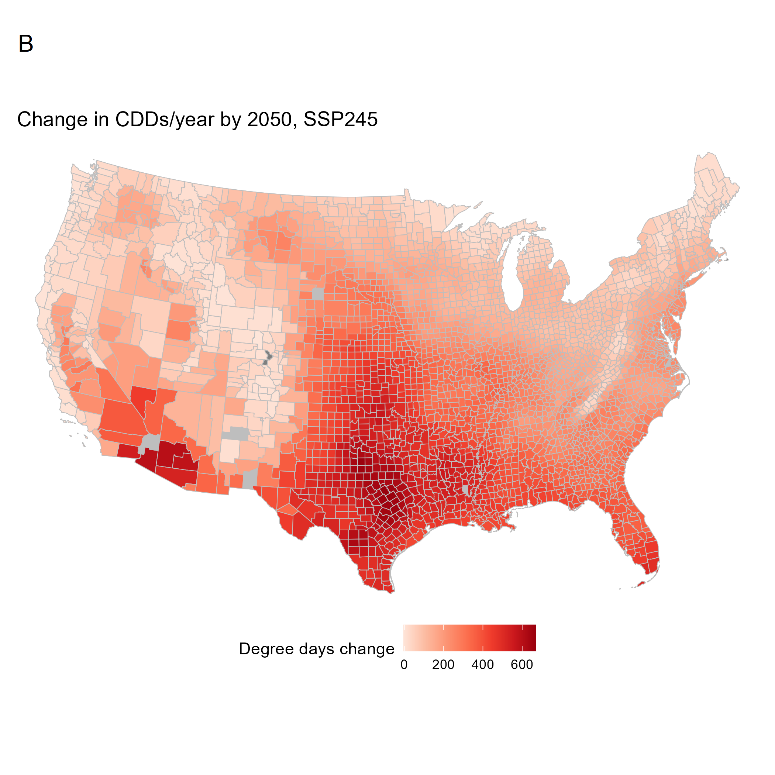


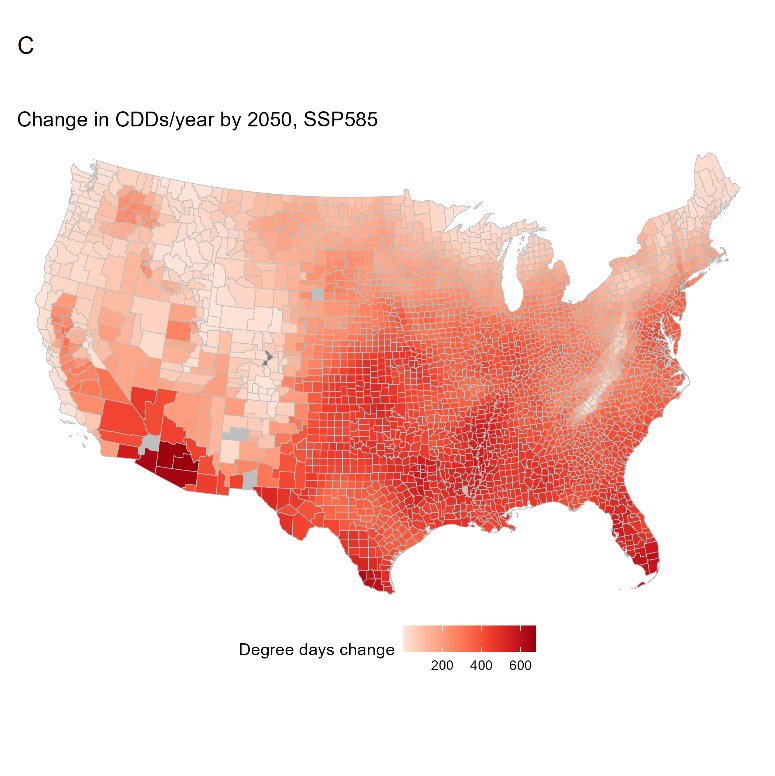


Supplementary Figures 3A to 3C. 95th Percentile of 20-Year Daily Maximum Temperatures (TMax95) in Early 21st Century (1995-2014) and Projected Change by 2050, Based on SSP245 and SSP585 Assumptions


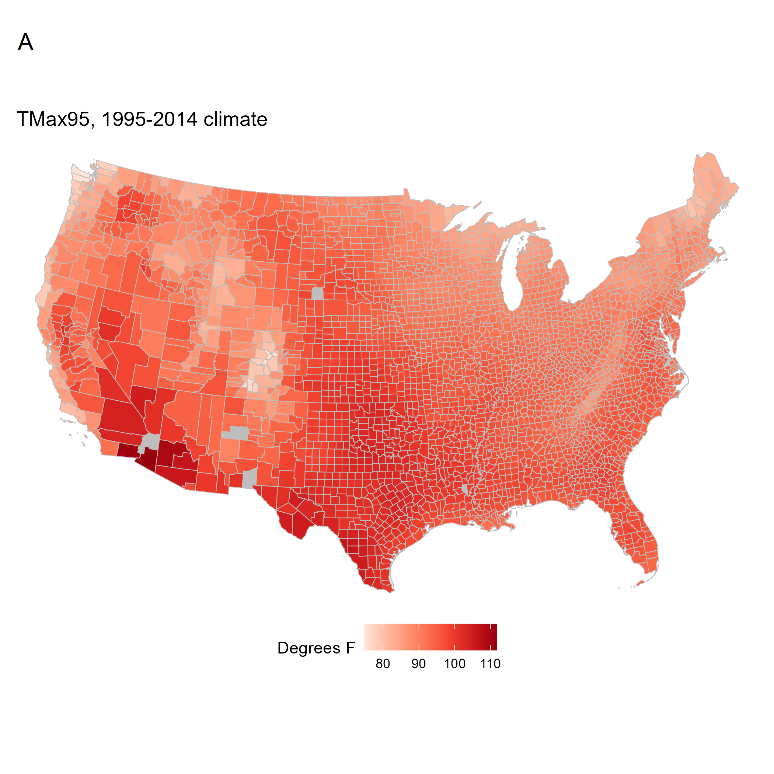

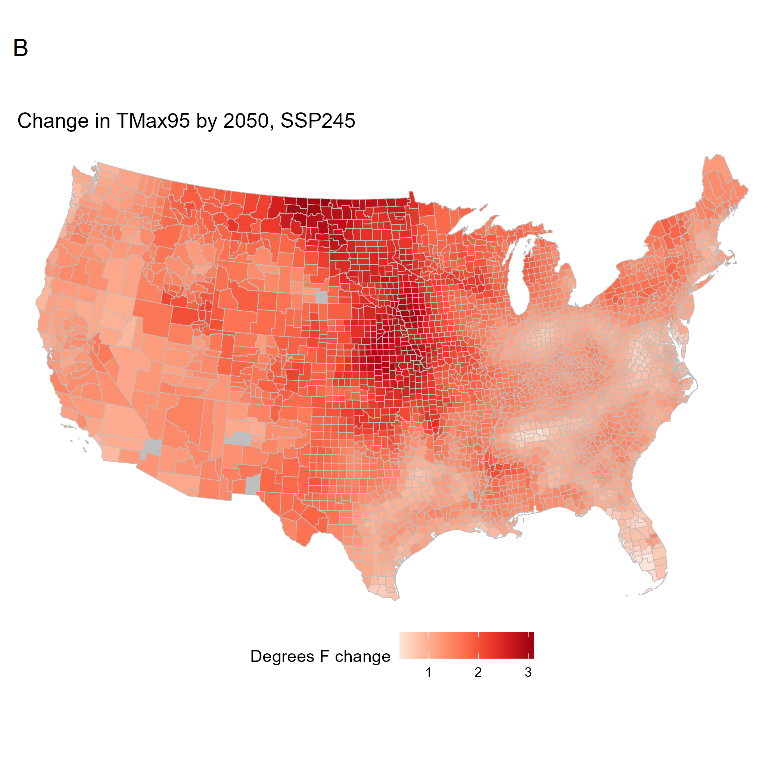


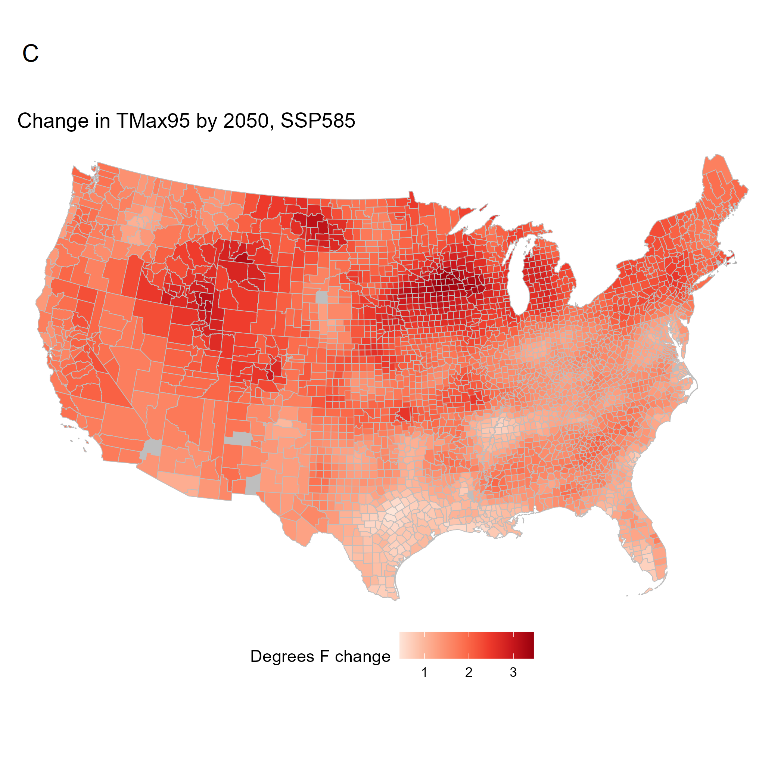


Supplementary Figure 4A to 4D. Percentage of Population Aged 69+, Annual Cooling Degree Days (CDDs), and 95th Percentile of 20-Year daily Maximum Temperatures, Contemporary Indicators (1995-2014) and Projected Change by 2050, Based on SSP245 Assumptions

|  | Population and CDDs | Population and TMax95 |
| --- | --- | --- |
|  | 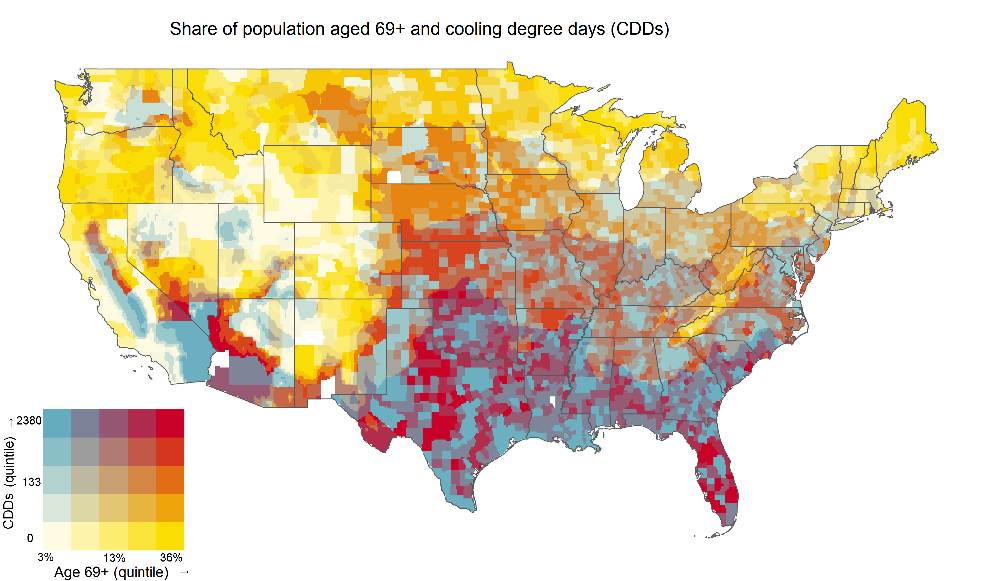  A | 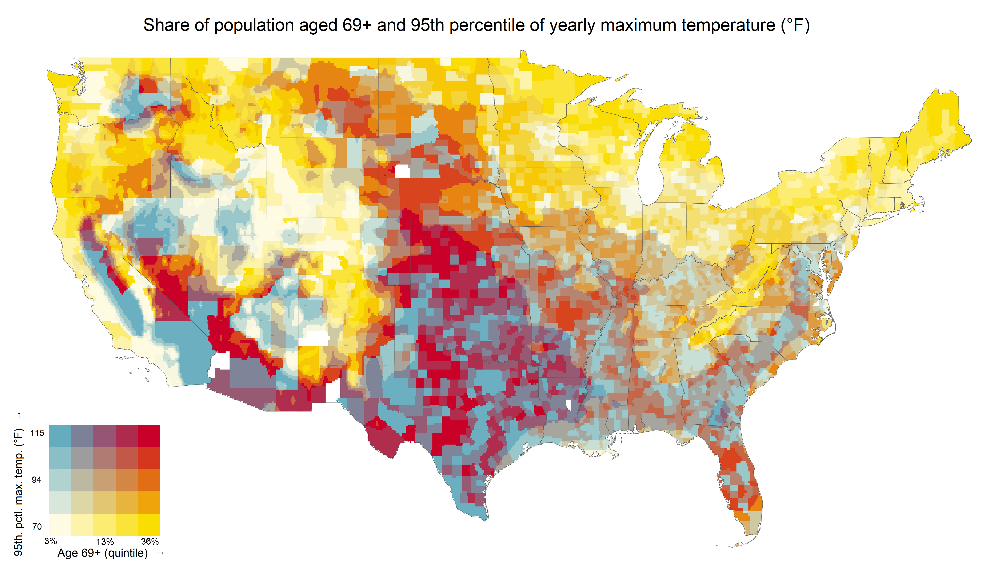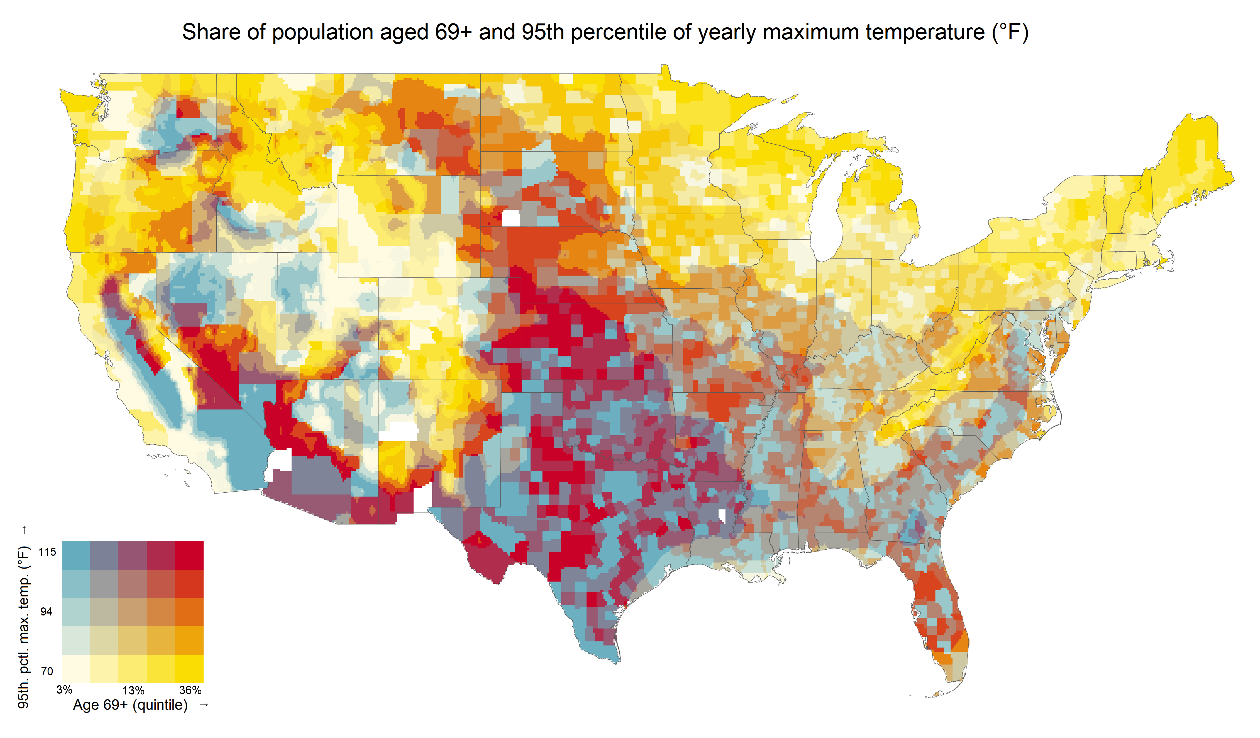  B |
|  | 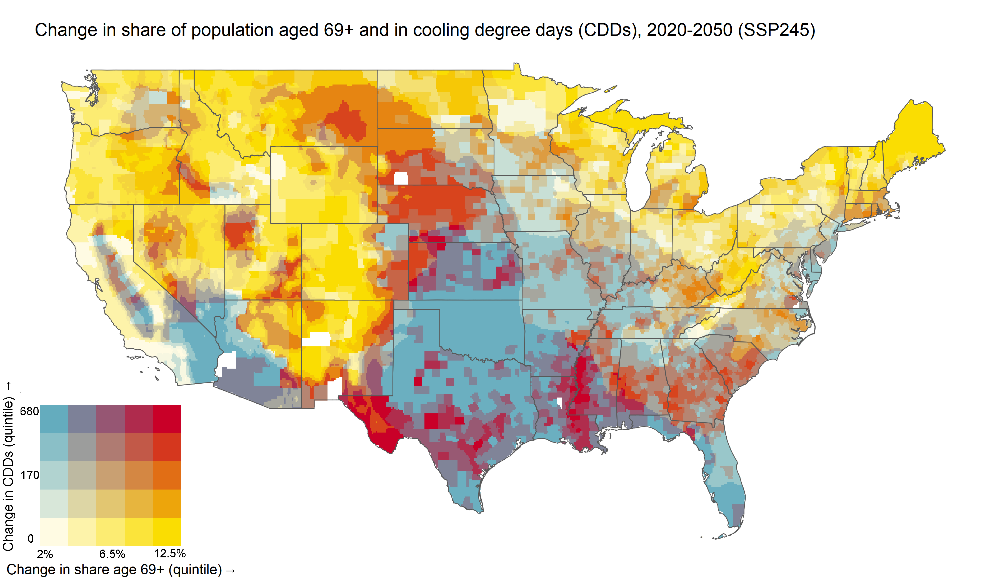  C | 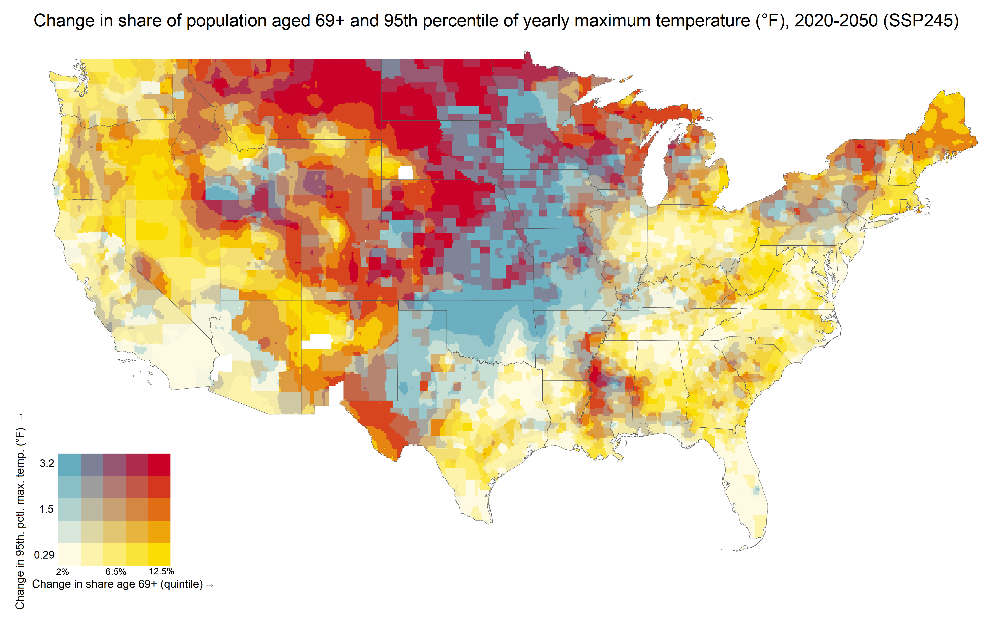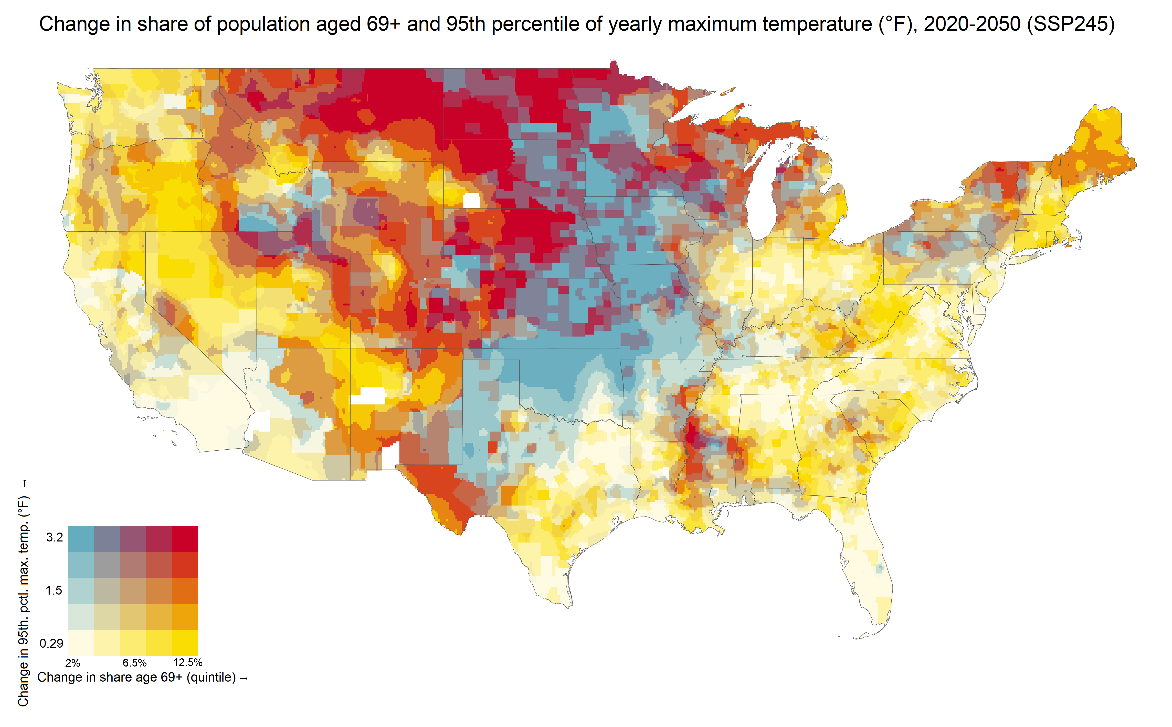  D |

Supplementary Figure 5. Relative Contributions of Climate Change, Population Growth, and Population Aging to Person Cooling Degree Days, by 48 Conterminous U.S. States, 2050

**
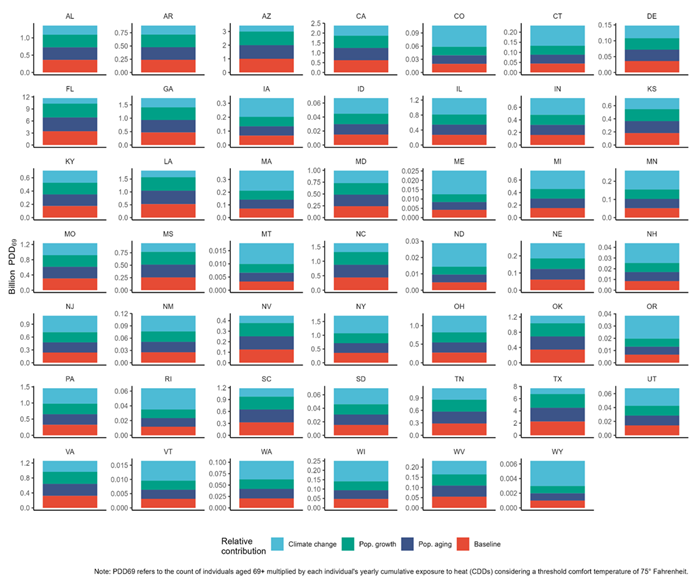
**
